# Implementing an Evidence-Based Guideline to Improve Perioperative Antimicrobial Prophylaxis Quality Among Patients With Penicillin Allergy Labels

**DOI:** 10.1016/j.mayocpiqo.2026.100724

**Published:** 2026-06-01

**Authors:** Sarah Lessard, Alexei Gonzalez-Estrada, Steven B. Porter, Michael A. Edwards, Natalie Hamilton, Kelli Andraschko, Kalla Evans, Lindsey Sangaralingham, Jennifer L. Ridgeway, Douglas Challener, Ebrahim H. Khan, Viengneesee Thao, Sarah Welle, Alejandra Villavicencio, Yu-Hui Chang, Abinash Virk

**Affiliations:** a Department of Pharmacy, Mayo Clinic Health System, La Crosse, WI; b Division of Allergy, Asthma, & Clinical Immunology, Mayo Clinic, Scottsdale, AZ; c Department of Anesthesiology and Perioperative Medicine, Mayo Clinic, Jacksonville, FL; d Division of Advanced GI and Bariatric Surgery, Mayo Clinic, FL; e Center for Individualized Medicine, Mayo Clinic, Rochester, MN; f Robert D. and Patricia E. Kern Center for the Science of Healthcare Delivery, Mayo Clinic, Rochester, MN; g Division of Infectious Disease, Mayo Clinic, Rochester, MN; h Metro Infectious Disease Consultants, Burr Ridge, IL; i Clinical Informatics & Practice Support, Mayo Clinic, Rochester, MN; j Department of Infection Prevention & Control, Mayo Clinic, Rochester, MN

## Abstract

**Objective:**

To implement a quality improvement project to promote cefazolin for surgical antimicrobial prophylaxis (SAP) among patients with penicillin allergy labels (PwPAL) as updated guidelines suggest shifting SAP to preferred antibiotics (cefazolin) in PwPAL, including penicillin-associated anaphylaxis.

**Patients and Methods:**

An interdisciplinary quality improvement project was conducted in a large, multiregion health care system in the United States to promote cefazolin for SAP among PwPAL. Interventions occurred January 1, 2023 through November 30, 2023 and were applied to adult and pediatric PwPAL undergoing operation. Broad system-based changes were implemented, including electronic health record allergy module enhancements, algorithm development, and point-of-care guidance to surgical clinicians through modifications to the electronic health record allergy-antibiotic flag at order entry and to surgical order sets. A dashboard was created to capture all SAP data, and education modules and communication articles were disseminated. Practice representative input was obtained, and frequent updates were provided through departmental meetings.

**Results:**

Use of cefazolin for SAP in PwPAL improved 38% across all regions within our health care organization, increasing from 63.4% (8349/13,164) to 89.1% (12,988/14,569), *P*≤.001. No difference in use of medications for hypersensitivity or tryptase testing was noted between the preintervention cohort and postintervention cohort. Reductions in clindamycin, from 9.1% (1198/13,164) to 0.7% (100/14,569), *P*≤.001), and vancomycin, from 16.7% (2194/13,164) to 4.7% (691/14,569), *P*<.001) were also noted.

**Conclusion:**

A successful increase in the use of cefazolin for SAP in PwPAL was achieved through broad quality improvement project implementation of system-based interventions without a corresponding increase in hypersensitivity surrogate markers.

Historically, cephalosporins, such as cefazolin, have been avoided for surgical antimicrobial prophylaxis (SAP) in patients with penicillin allergy labels (PwPAL), including penicillin-associated anaphylaxis, to the detriment of patients for concern for possible cross-reaction.^1^ Cefazolin is recommended for SAP for numerous surgical procedures, given its spectrum of coverage, bactericidal activity, and favorable pharmacokinetic properties but current data reports that most PwPAL receive alternative, nonpreferred, second-line, perioperative antibiotics, such as vancomycin and clindamycin, for SAP.^1^ Use of such alternative, nonpreferred SAP is associated with numerous safety concerns, including a 50% increased odds of surgical site infections (SSIs) and higher risk of adverse effects, including increased risk of resistance, and acute kidney injury.^1^, ^2^, ^3^, ^4^, ^5^, ^6^ Non β-lactam alternative antibiotics are also associated with drawbacks of prolonged infusion times and greater cost.^2^, ^3^, ^4^, ^5^, ^6^

Updated allergy guidelines suggest the use of dissimilar R1 side chain cephalosporin antibiotics, such as cefazolin, in PwPAL, including those with penicillin-associated anaphylaxis, is safe and without the historically perceived increased risk of hypersensitivity reactions.^7^ As such, the Society for Healthcare Epidemiology of America, the Infectious Diseases Society of America, the Association for Professionals in Infection Control and Epidemiology, the American Hospital Association, and The Joint Commission SSI preventive guidelines recommend improving use of β-lactam antibiotics for SAP in PwPAL.^8^ On the basis of this recommendation, select individual regions within our health care system successfully implemented efforts to promote cefazolin use for SAP in PwPAL without noted safety events.^9^^,^^10^ Efforts to expand and optimize SAP via a formal quality improvement project (QIP) to all regions within our health care organization to was initiated to improve patient outcomes, safety, and reduce cost. The aim of this QIP was to implement guidelines and improve cefazolin utilization for SAP in PwPAL, including those with penicillin-associated anaphylaxis to ≥80% across all regions within our health care organization without a corresponding increase in the rate of hypersensitivity or anaphylaxis.

## Methods

### Context

An interdisciplinary QIP was conducted in a large, multiregion health care system with 3 tertiary medical centers (regions 1, 2, and 3) and 4 regional health care systems (regions 4, 5, 6, and 7) within the United States. The Define, Measure, Analyze, Improve, Control framework was used to guide the design, implementation, and evaluation of this quality improvement initiative.^11^ A project charter, for resource allocation, was developed and approved. A project manager oversaw daily project activities. In 2022, this health care system performed 129,417 same-day surgical procedures in operating rooms with 15,636 same-day surgical procedures occurring in PwPAL for a variety of surgical subspecialties (see Table 1). Interventions occurred in January 2023 through November 2023. Data measurements included the following 3 cohorts: (1) preintervention cohort from January 2022 to December 2022, (2) intervention cohort from January 2023 to December 2023, including a washout period, and (3) postintervention cohort from January 2024 to December 2024. Interventions were applied to adult and pediatric PwPAL undergoing operation where SAP was ordered. This QIP was deemed exempt from review by the institutional review board.

**Table 1:** Cohort Characteristics, n (%)^a^

Characteristic	Year				
	2022 (n=15,636)	2023 (n=16,457)	2024 (n=16,968)	Total (n=49,061)	*P*
Age, y					.01^b^
Mean ± SD	58.9 ± 19.02	59.2 ± 18.89	59.4 ± 18.81	59.2 ± 18.91	
Median (IQR)	63.0 (49.0-72.0)	64.0 (49.0-73.0)	64.0 (49.0-73.0)	64.0 (49.0-73.0)	
Age group, y					.09^c^
<18	667 (4.3)	680 (4.1)	668 (3.9)	2015 (4.1)	
18-54	4391 (28.1)	4556 (27.7)	4669 (27.5)	13,616 (27.8)	
55-64	3156 (20.2)	3230 (19.6)	3266 (19.2)	9652 (19.7)	
65+	7418 (47.4)	7984 (48.5)	8361 (49.3)	23,763 (48.4)	
Sex					.24^c^
Female	10,044 (64.2)	10,479 (63.7)	10,713 (63.1)	31,236 (63.7)	
Male	5585 (35.7)	5967 (36.3)	6248 (36.8)	17,800 (36.3)	
Race					.07^c^
White	14,717 (94.1)	15,507 (94.2)	15,893 (93.7)	46,117 (94.0)	
Black/African Ancestry	355 (2.3)	335 (2.0)	357 (2.1)	1047 (2.1)	
Asian	220 (1.4)	211 (1.3)	282 (1.7)	713 (1.5)	
American Indian/Alaskan Native	75 (0.5)	87 (0.5)	101 (0.6)	263 (0.5)	
Native Hawaiian/Other Pacific Islander	11 (0.1)	9 (0.1)	14 (0.1)	34 (0.1)	
Other/Missing	258 (1.7)	308 (1.9)	321 (1.9)	887 (1.8)	
Ethnicity					.03^c^
Not Hispanic	14,831 (94.9)	15,542 (94.4)	15,994 (94.3)	46,367 (94.5)	
Hispanic	524 (3.4)	547 (3.3)	617 (3.6)	1688 (3.4)	
Missing	281 (1.8)	368 (2.2)	357 (2.1)	1006 (2.1)	
Elixhauser comorbid conditions					
Alcohol abuse	463 (3.0)	478 (2.9)	541 (3.2)	1482 (3.0)	.28^c^
Cardiac arrhythmias	4032 (25.8)	4194 (25.5)	4347 (25.6)	12,573 (25.6)	.83^c^
Blood loss anemia	160 (1.0)	152 (0.9)	187 (1.1)	499 (1.0)	.27^c^
Congestive heart failure	1154 (7.4)	1338 (8.1)	1468 (8.7)	3960 (8.1)	<.001^c^
Chronic pulmonary disease	3011 (19.3)	3242 (19.7)	3354 (19.8)	9607 (19.6)	.46^c^
Coagulopathy	1010 (6.5)	1140 (6.9)	1190 (7.0)	3340 (6.8)	.11^c^
Anemia deficiency	828 (5.3)	1022 (6.2)	1104 (6.5)	2954 (6.0)	<.001^c^
Depression	2801 (17.9)	2835 (17.2)	2862 (16.9)	8498 (17.3)	.04^c^
Diabetes without chronic complications	2286 (14.6)	2456 (14.9)	2652 (15.6)	7394 (15.1)	.03^c^
Diabetes with chronic complications	1935 (12.4)	1983 (12.0)	2197 (12.9)	6115 (12.5)	.04^c^
Drug abuse	383 (2.4)	393 (2.4)	443 (2.6)	1219 (2.5)	.40^c^
Fluid and electrolyte disorders	2162 (13.8)	2374 (14.4)	2426 (14.3)	6962 (14.2)	.27^c^
AIDS/HIV	19 (0.1)	19 (0.1)	15 (0.1)	53 (0.1)	.62^c^
Hypertension, complicated	2766 (17.7)	3031 (18.4)	3201 (18.9)	8998 (18.3)	.02^c^
Hypertension, uncomplicated	6242 (39.9)	6603 (40.1)	6862 (40.4)	19,707 (40.2)	.63^c^
Hypothyroidism	2710 (17.3)	2891 (17.6)	3002 (17.7)	8603 (17.5)	.69^c^
Liver disease	1086 (6.9)	1174 (7.1)	1293 (7.6)	3553 (7.2)	.05^c^
Lymphoma	256 (1.6)	314 (1.9)	313 (1.8)	883 (1.8)	.16^c^
Metastatic cancer	992 (6.3)	1078 (6.6)	1073 (6.3)	3143 (6.4)	.65^c^
Obesity	5265 (33.7)	5123 (31.1)	5223 (30.8)	15,611 (31.8)	<.001^c^
Other neurological disorders	1041 (6.7)	1029 (6.3)	1044 (6.2)	3114 (6.3)	.14^c^
Paralysis	159 (1.0)	194 (1.2)	175 (1.0)	528 (1.1)	.29^c^
Peptic ulcer disease	173 (1.1)	182 (1.1)	219 (1.3)	574 (1.2)	.20^c^
Peripheral vascular disease	1846 (11.8)	1714 (10.4)	1767 (10.4)	5327 (10.9)	<.001^c^
Psychoses	47 (0.3)	75 (0.5)	63 (0.4)	185 (0.4)	.08^c^
Pulmonary circulation disorder	499 (3.2)	604 (3.7)	595 (3.5)	1698 (3.5)	.06^c^
Renal failure	2522 (16.1)	2635 (16.0)	2835 (16.7)	7992 (16.3)	.18^c^
Rheumatoid arthritis/collagen	1093 (7.0)	1248 (7.6)	1315 (7.7)	3656 (7.5)	.02^c^
Solid tumor without metastasis	3383 (21.6)	3658 (22.2)	3825 (22.5)	10,866 (22.1)	.14^c^
Valvular disease	1657 (10.6)	1798 (10.9)	1881 (11.1)	5336 (10.9)	.36^c^
Weight loss	701 (4.5)	780 (4.7)	790 (4.7)	2271 (4.6)	.5r^c^
Elixhauser sum of comorbid conditions					.23^b^
Mean ± SD	3.4 ± 3.01	3.4 ± 3.03	3.4 ± 3.05	3.4 ± 3.03	
Median (IQR)	3.0 (1.0-5.0)	3.0 (1.0-5.0)	3.0 (1.0-5.0)	3.0 (1.0-5.0)	
ASA physical status classification					<.001^c^
I	1101 (7.0)	991 (6.0)	945 (5.6)	3037 (6.2)	
II	6382 (40.8)	6339 (38.5)	6373 (37.6)	19,094 (38.9)	
III	6553 (41.9)	7384 (44.9)	7880 (46.4)	21,817 (44.5)	
IV	496 (3.2)	559 (3.4)	553 (3.3)	1608 (3.3)	
V	2 (0.0)	4 (0.0)	3 (0.0)	9 (0.0)	
Missing	1102 (7.0)	1180 (7.2)	1214 (7.2)	3496 (7.1)	
Wound classification					.09^c^
Clean	10,399 (66.5)	10,755 (65.4)	11,203 (66.0)	32,357 (66.0)	
Clean contaminated	5237 (33.5)	5702 (34.6)	5765 (34.0)	16,704 (34.0)	
Inpatient/outpatient					.18^c^
Outpatient operation	10,685 (68.3)	11,273 (68.5)	11,747 (69.2)	33,705 (68.7)	
Outpatient in a bed (outpatient overnight)	1943 (12.4)	1990 (12.1)	1964 (11.6)	5897 (12.0)	
Operation admit	3008 (19.2)	3194 (19.4)	3257 (19.2)	9459 (19.3)	
Surgical specialty					
Advanced GI and Bariatrics	77 (0.5)	130 (0.8)	161 (0.9)	368 (0.8)	<.001^c^
Anesthesiology	72 (0.5)	108 (0.7)	85 (0.5)	265 (0.5)	.04^c^
Breast and Melanoma Surgical Oncology	269 (1.7)	260 (1.6)	268 (1.6)	797 (1.6)	.52^c^
Cardiothoracic operation	161 (1.0)	151 (0.9)	175 (1.0)	487 (1.0)	.49^c^
Cardiovascular operation	261 (1.7)	271 (1.6)	269 (1.6)	801 (1.6)	.82^c^
Colon and Rectal operation	218 (1.4)	258 (1.6)	252 (1.5)	728 (1.5)	.44^c^
Dentistry	0 (0.0)	0 (0.0)	3 (0.0)	3 (0.0)	.06^c^
Dermatology	5 (0.0)	5 (0.0)	0 (0.0)	10 (0.0)	.07^c^
Electrophysiology	1 (0.0)	1 (0.0)	3 (0.0)	5 (0.0)	.49^c^
Endocrine operation	0 (0.0)	18 (0.1)	177 (1.0)	195 (0.4)	<.001^c^
Endocrine and Metabolic operation	291 (1.9)	215 (1.3)	0 (0.0)	506 (1.0)	<.001^c^
Family Medicine	1 (0.0)	0 (0.0)	0 (0.0)	1 (0.0)	.34^c^
Gastroenterology	22 (0.1)	15 (0.1)	2 (0.0)	39 (0.1)	<.001^c^
General operation	827 (5.3)	808 (4.9)	713 (4.2)	2348 (4.8)	<.001^c^
General operation Pediatrics	61 (0.4)	58 (0.4)	57 (0.3)	176 (0.4)	.71^c^
Gynecologic Oncology	178 (1.1)	182 (1.1)	187 (1.1)	547 (1.1)	.94^c^
Gynecologic operation	347 (2.2)	384 (2.3)	400 (2.4)	1131 (2.3)	.68^c^
Hematology Oncology	1 (0.0)	2 (0.0)	0 (0.0)	3 (0.0)	.36^c^
Hepatobiliary and Pancreas operation	169 (1.1)	211 (1.3)	195 (1.1)	575 (1.2)	.23^c^
Metabolic and Abdominal Wall Reconstructive operation	0 (0.0)	19 (0.1)	127 (0.7)	146 (0.3)	<.001^c^
Minimally Invasive Gynecologic operation	117 (0.7)	128 (0.8)	134 (0.8)	379 (0.8)	.91^c^
Neurological operation	956 (6.1)	976 (5.9)	1104 (6.5)	3036 (6.2)	.08^c^
Obstetrics and Gynecology	516 (3.3)	510 (3.1)	513 (3.0)	1539 (3.1)	.34^c^
Ophthalmology	2202 (14.1)	2163 (13.1)	2342 (13.8)	6707 (13.7)	.04^c^
Ophthalmology Retina	0 (0.0)	0 (0.0)	4 (0.0)	4 (0.0)	.02^c^
Oral and Maxillofacial operation	155 (1.0)	167 (1.0)	148 (0.9)	470 (1.0)	.36^c^
Orthopedic Surgery	3541 (22.6)	3703 (22.5)	3587 (21.1)	10,831 (22.1)	<.001^c^
Orthopedic operation hand	96 (0.6)	136 (0.8)	151 (0.9)	383 (0.8)	.01^c^
Orthopedic operation podiatry	224 (1.4)	259 (1.6)	233 (1.4)	716 (1.5)	.29^c^
Orthopedic operation spine	124 (0.8)	147 (0.9)	146 (0.9)	417 (0.8)	.61^c^
Otorhinolaryngology head and neck operation	1322 (8.5)	1453 (8.8)	1523 (9.0)	4298 (8.8)	.23^c^
Pain medicine	2 (0.0)	8 (0.0)	22 (0.1)	32 (0.1)	<.001^c^
Pediatric gastroenterology and hepatology	0 (0.0)	1 (0.0)	1 (0.0)	2 (0.0)	.63^c^
Pediatric orthopedic operation	10 (0.1)	9 (0.1)	18 (0.1)	37 (0.1)	.19^c^
Plastic operation	659 (4.2)	625 (3.8)	672 (4.0)	1956 (4.0)	.16^c^
Pulmonary medicine	16 (0.1)	32 (0.2)	38 (0.2)	86 (0.2)	.02^c^
Reproductive endocrinology and infertility	22 (0.1)	34 (0.2)	23 (0.1)	79 (0.2)	.20^c^
Surgical oncology	145 (0.9)	189 (1.1)	189 (1.1)	523 (1.1)	.12^c^
Surgical oncology and endocrine operation	0 (0.0)	1 (0.0)	194 (1.1)	195 (0.4)	<.001^c^
Surgical oncology and endocrine operation surgical oncology	140 (0.9)	147 (0.9)	9 (0.1)	296 (0.6)	<.001^c^
Thoracic operation	186 (1.2)	171 (1.0)	159 (0.9)	516 (1.1)	.08^c^
Transplant heart	3 (0.0)	2 (0.0)	3 (0.0)	8 (0.0)	.87^c^
Transplant liver kidney pancreas	233 (1.5)	256 (1.6)	239 (1.4)	728 (1.5)	.54^c^
Transplant lung	4 (0.0)	5 (0.0)	2 (0.0)	11 (0.0)	.50^c^
Trauma operation	97 (0.6)	99 (0.6)	127 (0.7)	323 (0.7)	.20^c^
Urogynecology	70 (0.4)	86 (0.5)	67 (0.4)	223 (0.5)	.22^c^
Urology	1534 (9.8)	1790 (10.9)	1946 (11.5)	5270 (10.7)	<.001^c^
Vascular operation	287 (1.8)	252 (1.5)	284 (1.7)	823 (1.7)	.11^c^

a Abbreviations: ASA, American Society of Anesthesiologists; IQR, interquartile range; SD, standard deviation.

b Kruskal-Wallis *P*-value.

c Chi-Square *P*-value.

### Interventions and Strategies

Practice representative engagement was sought and included 14 teams and committees, including members from Surgical and procedural practice, anesthesiology, allergy & immunology, surgical nursing, antimicrobial stewardship (AS), pharmacists, nursing and pharmacy informatics, infection prevention and control, and nursing, medical, and pharmacy education. Antimicrobial stewardship programs were active in all regions before this QIP and AS representatives were engaged in this QIP. No conflicting AS projects were observed during this QIP. Through structured brainstorming sessions with practice representatives, potential causes of nonpreferred preoperative antibiotics use among PwPAL were identified:

• Lack of awareness among prescribers regarding penicillin and cephalosporin cross reactivity rates.
• Absence of effective point-of-care tools, including inaccurate electronic health record (EHR) flagging of allergies and antibiotic orders.
• Lack of updated surgical order sets with appropriate guidance for best practice.
• Limited tools for identifying practice gaps and monitoring progress

On the basis of impact-effort grid assessments, broad system-based changes were implemented. A consensus-based algorithm and guideline to promote cefazolin as SAP in PwPAL, including penicillin-associated anaphylaxis, was developed (Supplemental Figure 1, available online at http://www.mcpiqojournal.org). An electronic dashboard was created to capture SAP for all surgical patients. This dashboard was the source for process metrics and was able to filter by multiple criteria, including surgical details, beta-lactam allergy, and methicillin-resistant *Staphylococcus aureus* status.

`Educational modules were developed to address the issue of limited awareness. Modules provided an evidence-based rationale for this practice change and included the details of updated clinical recommendations. Didactic modules were deployed through an online education platform to surgical and anesthesiology clinicians, including staff physicians, learners, advanced practice providers, pharmacists, and nursing teams with a one-time completion requirement with an estimated completion time of <10 minutes. The completion rates were tracked, and the modules remained accessible thereafter as a reference resource. Practice representatives’ input was sought initially and periodically thereafter. Regular (eg, quarterly), updates regarding project progress and implementation timelines were provided to practice representatives by health care system departmental meetings across all regions.

On the basis of information gathered, the EHR allergy module was updated to enhance allergy documentation capabilities (July 2023). The drug-allergy flag was not modifiable in the EHR; hence guidance was provided at clinician antibiotic order entry when EHR allergy-antibiotic alert occurred (Supplemental Figure 2, available online at http://www.mcpiqojournal.org) to promote the use of cefazolin, or other cephalosporins as appropriate, in PwPAL. Additionally, 127 electronically available surgical order sets were edited to provide recommended evidence-based guidance for SAP in PwPAL (November 2023). A surgical order set is a standardized collection of evidence-based EHR point-of-care orders such as analgesics, antibiotics, or other medications that support perioperative management for a specific surgical procedure. These order sets typically include antimicrobial prophylaxis selection, dosing, and timing and are used to promote consistency, safety, and adherence to clinical guidelines across providers and sites. Other than promoting use of cefazolin in PwPAL, no institutional changes to SAP were implemented.

### Measures

#### **Patients**

We abstracted the following patient characteristics from the EHR. age at time of operation, sex, race, and ethnicity, Elixhauser comorbid conditions up to 1 year before surgical date, and American Society of Anesthesiologists (ASA) scores. Wound classification, location of operation (inpatient or outpatient) and surgical specialty were also collected. Wound classification was extracted from the EHR and reflects the wound classification documented by the surgical care team at the time of the index surgical encounter. The Elixhauser comorbidity index is a validated approach for identifying pre-existing comorbidities in hospital administrative data.^12^

#### **Process Improvement Measure**

The primary improvement measure was the percentage of PwPAL who received cefazolin for SAP. This measure was selected as cefazolin is the preferred SAP per national guidelines and institutional protocol for numerous clean (cefazolin monotherapy) and clean-contaminated cases (in conjunction with metronidazole in some operations). The aim of this QIP was to improve cefazolin utilization for SAP in PwPAL to ≥80% across all regions within our health care organization without a corresponding increase in the rate of hypersensitivity or anaphylaxis. Baseline cefazolin use varied substantially by region and a threshold of ≥80% was identified as a goal to account for meaningful practice variations and lack of clear allergy details on all patients, which could preclude use of cefazolin safely without additional patient assessment such as a review by Allergy and Immunology preoperatively. Surgical order set use rates were also assessed.

#### **Balancing Measure**

The rates of hypersensitivity and anaphylaxis were selected as balancing measures. Surrogate indicators for anaphylaxis were defined as the administration of medications commonly used in acute hypersensitivity management (systemic epinephrine, diphenhydramine, hydrocortisone, and famotidine) or by the ordering of serum tryptase testing. These surrogates have been used in a prior study as proxies for perioperative anaphylaxis.^13^ The EHR order database was queried for medication orders of famotidine, epinephrine, hydrocortisone, diphenhydramine, and the serum tryptase lab order on the same day of operation. The frequency of administration of each drug and request for laboratory serum tryptase level in each period was calculated.

#### **Statistical Analyses of Measures**

Two-sided χ^2^ tests were performed on each outcome. All statistical analyses were conducted using SAS v7.13, with statistical significance defined as *P*<.05. We used an interrupted time-series analysis to assess the effect of our intervention on rates of cefazolin use. We fit 3 segmented regression models to each time period: the preintervention year (2022), the intervention year (2023), and the postintervention year (2024).

We used the PROC GENMOD command in SAS with a binomial distribution, log link, and repeated statement to account for correlated data between regions. We tested for significant changes in slopes between the preintervention period and postintervention period and between the intervention period and the postintervention period.

### Analyses

Preintervention data assessment identified multiple regions were below goal for use of cefazolin for SAP in PwPAL. Use of cefazolin in PwPAL undergoing elective operation in 2022 was 8349/13,164 (63.4%) across all regions within our health care organization with regional sites variations: region 1 was 61.1%, region 2 was 45.7%, region 3 was 44.2%, region 4 was 90.3%, region 5 was 90.4%, region 6 was 78%, and region 7 was 80.6%. Higher preintervention utilization rates in nontertiary center regions 4-7 were attributable to penicillin allergy SAP process improvement efforts before this QIP.^9^^,^^10^ Surgical order set use, to order SAP, at preintervention was 88.8%.

Summary of input received from key practice representatives indicated the gap in quality was based on long-standing historical practice, and widespread system adjustments and education efforts would be imperative for project success.

## Results

**Figure 1:**
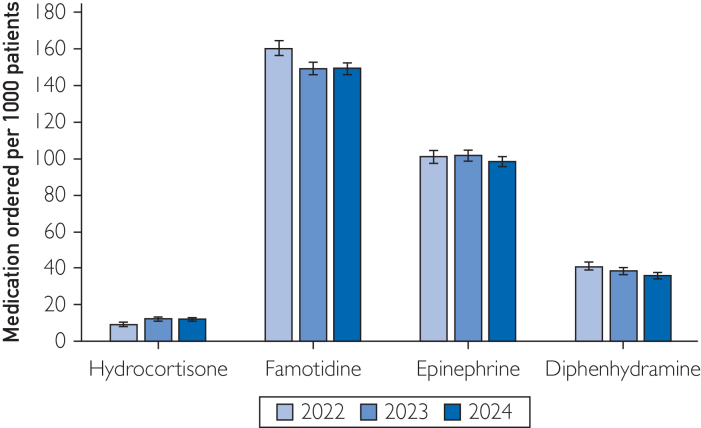
Medication use for hypersensitivity surrogate. The balancing measure, a surrogate for hypersensitivity (eg, anaphylaxis), was captured by use of medications (rate mediation ordered per 1000 patients) for hypersensitivity (hydrocortisone, famotidine, epinephrine, and diphenhydramine). No statistically significant differences were observed across years for any medication (χ^2^ test; all *P* >.05).

**Figure 2:**
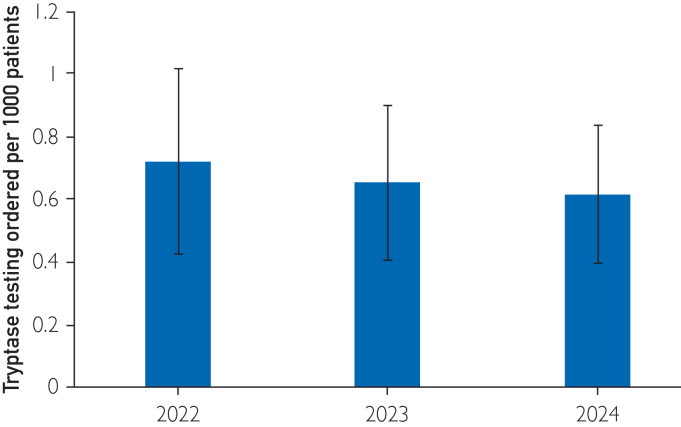
Tryptase orders for hypersensitivity surrogate. The balancing measure, a surrogate for anaphylaxis (hypersensitivity), was captured by use of tryptase testing (rate of testing ordered per 1000 patients) in patients with penicillin allergy who received cefazolin. Error bars represent standard error (χ^2^ test; *P*=.96).

**Figure 3:**
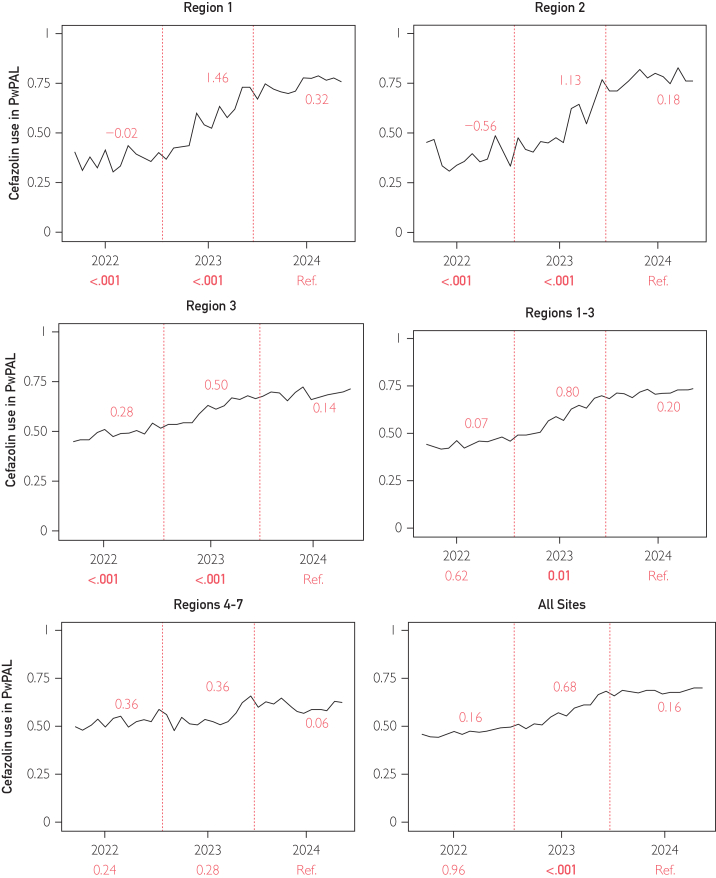
Time-series analysis for cefazolin use at preintervention (2022), intervention (2023), and postintervention (2024). The red numbers inside the graphs represented the slopes of the lines (rate of increase of cefazolin use), while the red numbers below the graphs indicated the *P* values. Higher slopes were observed for tertiary regions 1-3, as compared with nontertiary regions 4-7, which was expected as cefazolin utilization rates in nontertiary regions 4-7 were higher due to prior process improvement efforts. PwPAL, patients with penicillin allergy labels.

Cohort characteristics of age, gender, race, ethnicity, Elixhauser sum of comorbid conditions, surgical wound classification or inpatient/outpatient status did not differ significantly between the preintervention, intervention and postintervention periods (Table 1). The overall proportion of PwPAL was consistent from year to year with 12.3% in 2022, 12.3% in 2023 and 12.5% in 2024. Increased ASA scores were noted from 2022 to 2024 along with modest changes in surgical subspecialties completing procedures (Table 1). Use of cefazolin for SAP in PwPAL improved 38% across all regions within our health care organization, increasing from 63% (8349/13,164) to 89% (12,988/14,569), *P*≤.001 (Table 2). No difference in anaphylaxis surrogates (use of medications for hypersensitivity or serum tryptase testing) was noted between the preintervention cohort and postintervention cohort (Figures 1 and 2). Use of second-line antibiotics for SAP in PAL patients reduced significantly in the postimplementation cohort with reductions in clindamycin, from 9.1% (1198/13,164) to 0.7% (100/14,569), *P*≤.001) and vancomycin, from 16.7% (2194/13,164) to 4.7% (691/14,569), *P*<.001) noted (Table 2). A time-series analysis reported significant uptake for the use of cefazolin during the intervention year in tertiary center regions 1-3 (Figure 3). Surgical order set use to order SAP was similar at preintervention (88.8%) and postintervention (88.9%). Didactic education modules were completed by 99% (2869 of 2897) of clinicians.

**Table 2:** Improvement Measure

Region	Year	No Antibiotic for SAP (n)	Any Antibiotic for SAP (n)	Cefazolin (n, %)	Vancomycin (n, %)	Clindamycin (n, %)
All Regions	2022	4574	13164	8349 (63.4)	2194 (16.7)	1198 (9.1)
	2024	4213	14569	12,988 (89.1)	691 (4.7)	100 (0.7)
	*P*-value^a^	<.001	<0.001	<.001	<.001	<.001
Region 1	2022	1802	5948	3810 (61.1)	892 (15)	506 (8.5)
	2024	1970	6554	5892 (89.8)	273 (4.2)	27 (0.04)
	*P*-value^a^	.83	.83	<.001	<.001	<.001
Region 2	2022	356	1931	883 (45.7)	518 (26.8)	292 (15.1)
	2024	317	2298	2020 (87.9)	182 (7.9)	26 (1.1)
	*P*-value^a^	<.001	<.001	<.001	<.001	<.001
Region 3	2022	389	2064	913 (44.2)	623 (30.2)	300 (14.5)
	2024	361	2379	2037 (85.6)	168 (7.1)	32 (1.3)
	*P*-value^a^	.01	.01	<.011	<.001	<.001
Region 4	2022	466	1059	1059 (90.3)	29 (2.5)	14 (1.2)
	2024	554	1178	1178 (92.2)	10 (0.8)	4 (0.3)
	*P*-value^a^	.24	.24	.85	<.001	.01
Region 5	2022	716	522	472 (90.4)	5 (1)	1 (0.2)
	2024	402	581	515 (88.6)	0 (0)	0 (0)
	*P*-value^a^	<.001	<.001	<.001	.051	.37
Region 6	2022	499	667	520 (78)	68 (10.2)	48 (7.2)
	2024	420	650	602 (92.6)	24 (3.7)	6 (0.9)
	*P*-value^a^	.09	.09	<.001	<.001	<.001
Region 7	2022	346	859	692 (80.6)	59 (6.9)	37 (4.3)
	2024	299	829	744 (89.7)	34 (4.1)	5 (0.6)
	*P*-value^a^	.23	.23	<.001	.02	<.001

Use of cefazolin in patients with penicillin allergy labels undergoing operation detailed by all regions and individual regions preintervention (2022) and postintervention (2024).

SAP, surgical antimicrobial prophylaxis.

a Chi-Square *P*-value.

## Discussion

Successful increase in use of cefazolin for SAP in PwPAL was achieved through broad QIP implementation of system-based changes, including education to multiple disciplines, updates to EHR allergy-antibiotic alerts, and order set updates. In the interrupted time-series (Figure 3), the postintervention flattening of the slope suggests the system adopted the intervention and reached a new steady state of practice, with high performance observed for all regions.

Increased use of cefazolin for SAP in PwPAL was not associated with an increase in surrogate markers for hypersensitivity (eg, anaphylaxis) as assessed by the intra-operative use of medications for hypersensitivity treatment or serum tryptase testing. These findings compliment prior publications.^2^, ^3^, ^4^, ^5^, ^6^^,^^10^^,^^11^ The findings of this project report that implementing evidence-based guidelines for promoting cefazolin for SAP in PwPAL, including those with penicillin-associated anaphylaxis, is safe and supports a paradigm shift away from routine avoidance of cephalosporins in PwPAL. Clinicians can confidently prescribe cefazolin for SAP in most PwPALs, reducing unnecessary use of second-line, broad-spectrum antibiotics.

Nearly all patients with penicillin allergies can receive cefazolin for SAP safely. Avoidance of cephalosporins, such as cefazolin, in PwPAL is unnecessary for SAP, except for patients who have cefazolin allergy or drug fever, a history of anaphylaxis to a cephalosporin, or a severe cutaneous adverse reaction to either a penicillin or cephalosporin.^7^ At our large multilevel health care system (including community and tertiary referral centers), broad education, communication, and changes to system support were critical to the success of the project in improving cefazolin use for SAP in PwPALs.

We acknowledge the potential for confounding if the proportion of PwPAL for whom cefazolin was clinically indicated differed between the preintervention and postintervention periods. However, assessment of surgical specialty distribution (Table 1) reported only modest differences between cohorts, suggesting that changes in case mix alone are unlikely to fully explain the observed increase in cefazolin utilization. Cefazolin was the primary recommended SAP for most of our patients throughout the study and no major changes in SAP recommendations occurred during this timeframe, so we are confident that the increase in cefazolin use in PwPAL for SAP is due to our quality improvement efforts.

Regions 4-7 reported higher baseline performance than regions 1-3 before initiation of the health care system-wide QIP. These higher-performing regions were intentionally included because the initiative was designed as a health care system-wide quality improvement effort rather than a targeted remediation project focused solely on low-performing sites. Given the substantial inter-regional variation, inclusion of all regions allowed for evaluation of root causes and potential solutions at a system level. Importantly, higher-performing centers achieved their success through differing approaches, including education-only strategies and AS–supported prescribing. The inclusion of these regions enabled assessment of which implementation strategies were associated with higher performance and informed development of scalable, system-wide interventions.

Limitations. Our data does not differentiate reported reactions in PwPAL (eg, rash vs hives vs anaphylaxis vs severe cutaneous adverse reaction). Prior assessment in region 7 reported that 42% of PwPAL had reported anaphylaxis or hives therefore we estimate similar proportion of patients had anaphylaxis or hives.^10^ Regardless, national guidelines recommend use of cefazolin in PwPAL, including those with penicillin-associated anaphylaxis without additional testing or precautions.^7^

Our surrogate markers for anaphylaxis (hypersensitivity), including serum tryptase testing, and use of medications including famotidine, epinephrine, hydrocortisone, and diphenhydramine, could be considered nonspecific as these medications are administered perioperatively with regularity for nonhypersensitivity scenarios. For example, famotidine is administered for antacid effects, hydrocortisone may be administered for stress dose steroid use preoperatively and epinephrine may be administered for inotropy support. However, the incidence of use of these medications did not signal a concern for use for hypersensitivity nor anaphylaxis and did not differ in our cohorts (Figures 1 and 2).

Although patients with a known history of severe cutaneous adverse reactions (SCARs) to penicillin were excluded, we acknowledge that accurate identification of prior SCAR relies on clinical history and may be imperfect, particularly when assessed by non-allergists. As this study was not designed to systematically capture delayed hypersensitivity reactions, rare SCAR events related to prior penicillin use may not have been detected. However, SCARs to β-lactam antimicrobials are exceedingly uncommon, and no clinically apparent SCAR events were identified during the study period.

Possible limitations for other institutions mirroring this QIP include availability of EHR or of resources for project management, dashboard development, and EHR modifications. Each of these realms was time and resource intensive.

Lessons Learned. Lessons learned from this QIP include the importance of interdisciplinary staff and practice representative engagement. Practice representatives indicated that enhancements to the EHR would be required to optimize β-lactam antibiotic use in PwPAL, and without changes to the EHR that uptake would be limited. The need for frequent communications of project efforts through various interdisciplinary teams (surgical, anesthesia, pharmacy, nursing, and informatics) was vital to project success. To sustain improvement and to identify the need for additional interventions targeted to the unique needs of some teams/settings, monitoring of dashboard and adherence to SAP will be completed by the health care organization’s AS program and surgical and procedural leadership.

## Conclusion

Successful increase in use of cefazolin for SAP in PwPALs is achievable through implementation of several system-based changes, including education to multiple disciplines, and point-of-care tools such EHR allergy-antibiotic alerts and guideline concordant order sets.

## Potential Competing Interests

The authors report no competing interests.
