# Supplementary material for: Implementing an Evidence-Based Guideline to Improve Perioperative Antimicrobial Prophylaxis Quality Among Patients With Penicillin Allergy Labels
Source: Mayo Clin Proc Innov Qual Outcomes. 2026 Jun 1;10(4):100724. doi: 10.1016/j.mayocpiqo.2026.100724 (PMC13251490; doi:10.1016/j.mayocpiqo.2026.100724)
Supplement: Supplemental Figure [file mmc1.pdf]

**Supplemental Figure 1.**

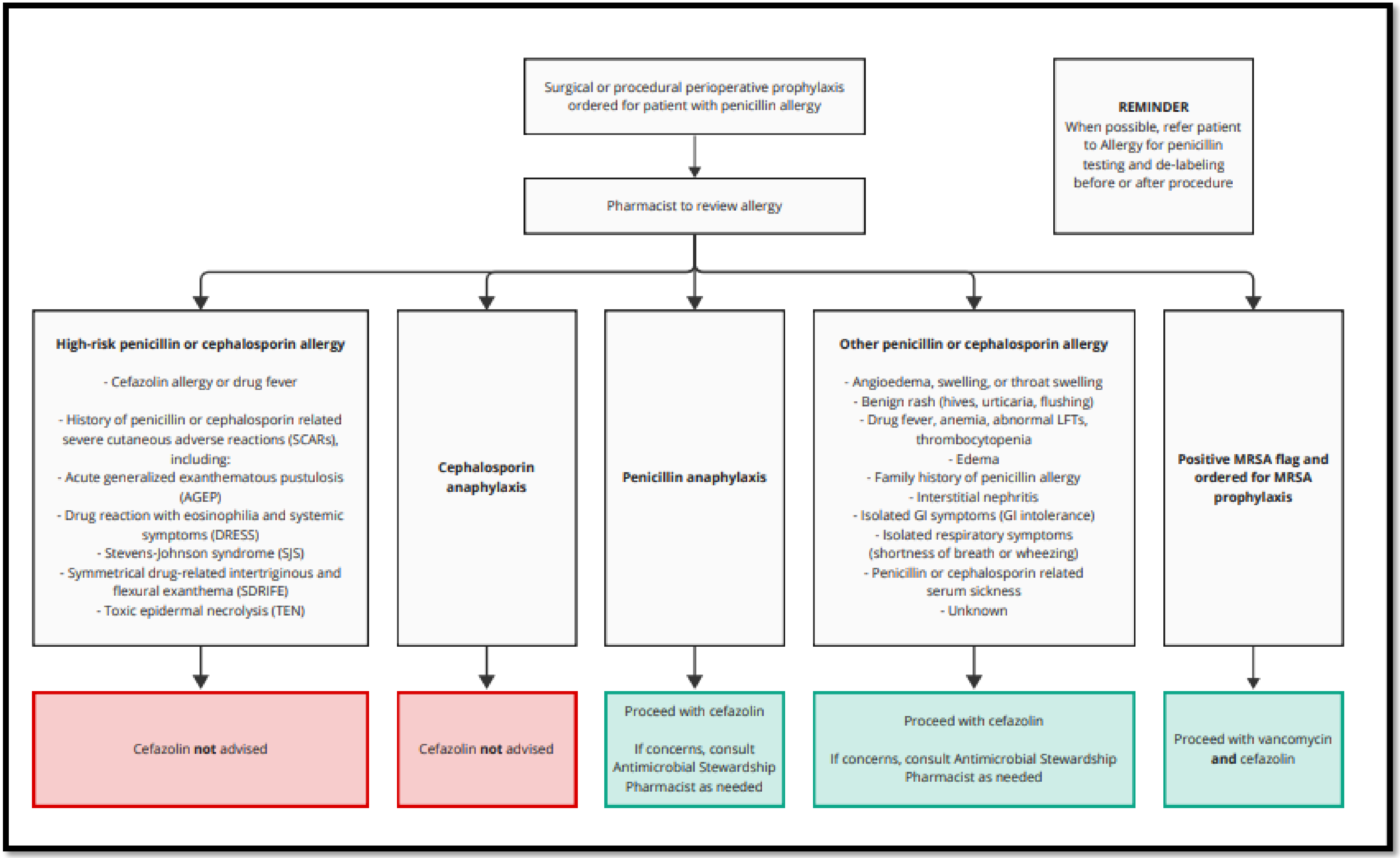


Algorithm to guide the use of cefazolin for surgical antimicrobial prophylaxis in patients with penicillin and cephalosporin allergies.

**Supplemental Figure 2.**

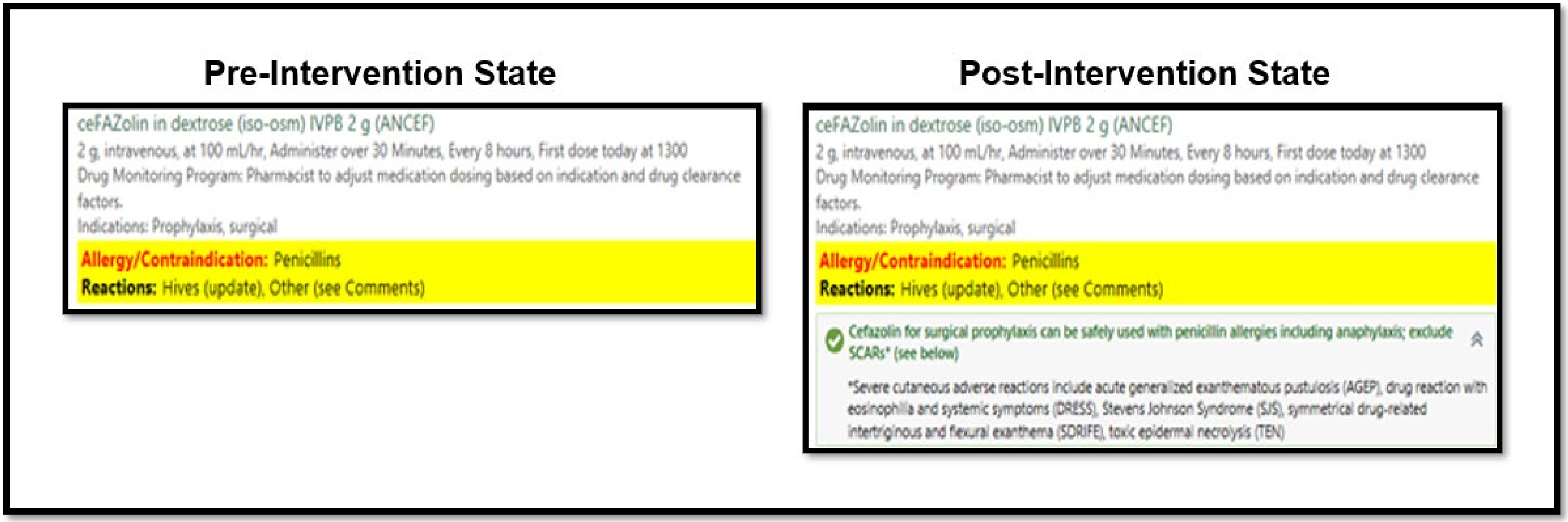


Electronic health record drug-allergy alert modification when cefazolin is ordered for surgical antimicrobial prophylaxis in patients with penicillin allergy label. © 2025 Epic Systems Corporation.
